# Comprehensive Pan-Cancer Analysis of RAC1 Decoding the Impact on Cancer Prognosis and B cell immune Regulation

**DOI:** 10.7150/ijms.104488

**Published:** 2025-01-13

**Authors:** Zhibo Shao, Hengyu Ren, Xuliren Wang, Qi Zhang, Jingyan Xue, Yayun Chi, Bingqiu Xiu, Jiong Wu

**Affiliations:** 1 Department of Breast Surgery, Key Laboratory of Breast Cancer in Shanghai, Fudan University Shanghai Cancer Center, Shanghai 200032, China.; 2 Department of Oncology, Shanghai Medical College, Fudan University, Shanghai 200032, China.; 3 Pathology Center, Shanghai General Hospital, Shanghai Jiao Tong University School of Medicine, Shanghai 200032, China.

## Abstract

RAC1, a member of the Rho family GTPases, has been implicated in various cancers, yet its pan-cancer landscape and role in the tumor immune microenvironment remain underexplored. This study presents a comprehensive analysis of RAC1 across 33 cancer types, revealing its high expression in a broad range of cancers and its association with poor prognosis. RAC1 expression correlates with genomic alterations, including CNVs, TMB, and MSI. RAC1 knockdown reduces cell proliferation and metastasis in breast and lung cancer cells, suggesting its oncogenic potential. Notably, RAC1 is negatively correlated with B cell infiltration, indicating its role in regulating the immune microenvironment. Functional enrichment analysis showed that high RAC1 expression is linked to lower enrichment in B cell activation and immune response pathways. Single-cell transcriptome analysis identified RAC1 expression primarily in epithelial cells, associated with tumor progression, and spatial transcriptome analysis showed a mutually exclusive co-localization between B cell infiltration regions and RAC1-expressing epithelial cells. Based on RAC1 expression and B cell interaction, a prognostic signature was established to predict prognosis at the pan-cancer level.

## Introduction

Cancer is a major contributor to global mortality, posing significant challenges to efforts aimed at extending human lifespan^1^. Advances in diagnostics have led to a surge in cancer detection, amplifying the global disease burden^2^. Breakthroughs in targeted therapies have significantly improved outcomes for several cancers^3^. Therefore, it is crucial to discover additional therapeutic targets and prognostic indicators for cancer.

RAC1, or Ras-related C3 botulinum toxin substrate 1, is a small GTPase protein that is a critical regulator of cell motility, cytoskeletal dynamics, oxidative stress response and inflammatory processes. In cancer, RAC1's overexpression is linked to tumor growth, metastasis, and resistance to chemotherapy, positioning it as a promising therapeutic target^4^. RAC1 is commonly characterized by its heightened expression and/or enhanced activity in a spectrum of solid malignancies, such as bladder^5^, and hepatocellular carcinomas^6^. At the same time, RAC1 is also closely related to tumor metastasis^7^. In lung cancer, a study has indicated that RAC1 is highly expressed in circulating tumor cells and lung metastatic cells^8^. In bladder cancer, the KDM6A-ARHGDIB axis can suppress the migratory ability of tumor cells by inhibiting RAC1^9^. Additionally, prior studies have indicated RAC1's role in the development of drug resistance across various cancer types^10^. In breast cancer, a study has demonstrated that RAC1 is associated with endocrine therapy resistance in estrogen receptor-positive breast cancer^11^. Moreover, within melanoma, RAC1 can enhance the expression of PDL1^12^. However, despite RAC1 being a potent biomarker for cancer, our understanding of its role in pan-cancers as well as immune microenvironment remodeling is not yet comprehensive. Therefore, there is an urgent need to investigate the function of RAC1 across various types of cancers.

Our study found that RAC1 is highly expressed in multiple cancers and associated with poor prognosis. It negatively correlates with B cell infiltration, suggesting a regulatory role in the immune microenvironment. Functional analysis linked high RAC1 expression to reduced activation of B cell-related pathways. Spatial transcriptomics revealed a mutually exclusive pattern between RAC1-expressing epithelial cells and B cell infiltration regions. We developed a prognostic signature combining RAC1 expression and B cell infiltration, which effectively predicts prognosis across cancer types.

## Materials and Methods

### Cancer data processing and analysis

All transcriptomic and clinical data encompassing 33 cancer types from The Cancer Genome Atlas (TCGA) data portal (https://portal.gdc.cancer.gov) are downloaded through the University of California Santa Cruz (UCSC) Xena platform (https://xena.ucsc.edu/)^13^. Single-cell transcriptomic data are obtained from the GEO database. These single-cell transcriptomic data of BRCA, LUAD, STAD, PAAD are derived from four GEO datasets (GSE161529^14^, GSE117570^15^, GSE167297^16^, GSE155698^17^) (https://www.ncbi.nlm.nih.gov/geo/)^18^. And These samples, comprising both normal tissue and tumour single-cell sequencing, have been selected for subsequent analysis. Spatial transcriptomics data for BRCA, LUAD, and PAAD were obtained from GSE225600^19^, GSE189487^20^, and GSE235315^21^, respectively. Additionally, RNA-seq data for LUAD were obtained from GSE30219^22^. The full names and abbreviations of the 33 cancer types explored can be found in Supplementary Table 1.

### Differential RAC1 expression analysis

The differential gene expression of RAC1 between normal tissues and tumor at pan-cancer level were analysed using the TIMER2.0 network server (http://timer.comp-genomics.org/)^23^ and the statistical significance was calculated using the Wilcoxon test. The variation in RAC1 mRNA expression between normal and tumour tissues in the GTEx and TCGA datasets was analysed with SangerBox. (http://sangerbox.com/)^24^ Furthermore, the expression of RAC1 in pan-cancer with corresponding normal tissues in the TCGA database was analysed by R software (version 4.3.1) and R package “ggpubr” (version 0.6.0). We calculated the differentially expressed significance with p value < 0.05 using one-way ANOVA.

### Analysis of the prognostic Role of RAC1

To explore the relationship between RAC1 expression and tumor prognosis at the pan-cancer level, we analyzed three prognostic indicators: Overall Survival (OS), Disease-Specific Survival (DSS), and Progression-Free Interval (PFI). This was achieved using univariate Cox regression analysis and Kaplan-Meier survival analysis with SangerBox. Samples are divided into high and low groups based on the median expression of RAC1. R packages “survminer” (version 0.4.9) and “survival” (version 3.5.5) were used to plot Kaplan-Meier survival curves and statistical significance was calculated by the log-rank test.

### Relationship between RAC1 expression and clinical stage

The relationship between RAC1 expression and clinical tumour stage in pan-cancer level was analysed using R package “ggpubr” (version 0.6.0). Significance was verified using Kruskal-Wallis test. Cancer types with significant p-values were selected and displayed using R packages “ggplot2” (version 3.4.4).

### Assessment of the diagnostic utility of RAC1

The diagnostic accuracy of RAC1, measured by the receiver operating characteristic (ROC) curve, area under the curve (AUC), sensitivity, and specificity, was evaluated. The analysis of the ROC curve was conducted using R package “pROC”^25^ (version 1.18.5). Typically, an AUC value close to 1.0 on the ROC curve signifies high diagnostic precision.

### Differential analysis and Enrichment analysis of RAC1

Samples in TCGA pan-cancer datasets are divided into high and low group according on the median expression of RAC1. Differentially expressed analysis for RAC1 in the pan-cancer level was performed by R package “DEseq2” (version 1.40.1). The DEGs in pan-cancer level were visualized by volcano plot using R package “ggplot2” (version 3.4.4). And top ten B cell related genes, such as CD19, CD79A, MS4A1, IGHG1 and so on, were selected to labelled on volcano plot. Furthermore, The DEGs in multi-type cancer were then conducted GSEA (Gene Set Enrichment Analysis enrichment) analyses on GO (Gene Ontology) pathway using R package “clusterProfiler” (version 4.8.1). Six GO pathway (“B cell activation”, “B cell receptor signalling pathway”, “immunoglobulin production”, “T cell receptor complex”, “immunoglobulin complex”, “antigen binding”) were defined as B cell activation and immune functions related pathway and selected to displayed using R package “ggplot2” (version 3.4.4). The significant GSEA enrichment results were determined on FDR, which is < 0.05.

### Correlation between RAC1 expression and Tumour Mutational Burden (TMB) and Microsatellite Instability (MSI)

All mutation-related information was sourced from the UCSC Xena and VarScan2^26^ platforms, with a focus on Variant Aggregation and Making data for detailed examination. The relationship between RAC1 mRNA expression and both Microsatellite Instability (MSI) and Tumour Mutational Burden (TMB) was assessed. The results were plotted as radar chart using R package “fmsb” (version 0.7.5). The interconnections between these variables were evaluated using Spearman's rank correlation coefficient analysis.

### Correlation between RAC1 expression and CNV levels

CNV (Copy number variation datasets) for all TCGA samples processed by the GISTIC^27^ (version 2.0) software form the GDC (https://portal.gdc.cancer.gov/). Copy number data and gene expression of RAC1 were integrated together. The integrated data were transformed each expression value with a log2(x+1) transformation. Finally, cancer types with fewer than three samples within a single cancer type were excluded. The visualization was performed by R package “ggplot2” (version 3.4.4).

### Correlation between RAC1 expression and immune cell infiltration

First, R package “CIBERSORT”^28^ (version 0.1.0) was used to compute immune cell infiltration score for 22 types of cell in the tumour immune microenvironment in pan-cancer level. Then, the expression levels of RAC1 and their correlation with these 22 types of tumour-infiltrating immune cells were calculated using Pearson correlation coefficient. An value of Pearson correlation coefficient greater than 0.15 is considered to indicate a positive correlation, while a value less than -0.15 indicates a negative correlation. The correlation results were displayed using a heatmap using R package “ComplexHeatmap” (version 2.16.0). R package “xCell”^29^ (version 1.1.0), “Timer”^30^ (version 2.0), as well as “Quantiseq”^31^ (version 1.10.0) were also used to infer the tumour immune infiltration in the pan-cancer levels. The B lineage score is equal to the sum of the fractions of B cell-related immune cell subsets in different R packages for inferring tumour immune infiltration. The Pearson correlation coefficient between the mRNA expression of RAC1 and B lineage score was calculated in pan-cancer levels including BRCA, LUAD, PAAD, STAD, BLCA and so on. The results are presented using scatter plots using R package “ggplot2” (version 3.4.4). ImmuneScore, StromalScore, and ESTIMATEScore for 33 different types of cancer in TCGA were conducted in R package “ESTIMATE”^32^ (version 1.0.13).

### Single cell sequencing analysis of RAC1

TISCH2 website (http://tisch.comp-genomics.org/home/)^33^ was used to examine the expression of RAC1 across different cellular components within the tumour microenvironment in the pan-cancer levels. Single-cell transcriptomic data downloaded from GEO were analysed in R using 10x matrix file in the R package “Seurat”^34^ (version 4.3.0). In brief, cells with > 20% of reads coming from mitochondrial transcripts and with < 300 nFeatures were excluded as probable dying cells and Contaminated cells. Batch effects were mitigated by employing R package “Harmony” (version 0.1.1). Additionally, R package “SingleR” (version 2.2.0) was utilized to assist the annotation of cell types within the clusters. All plots of single-cell transcriptomic data was drawn by functions in R package “Seurat” (version 4.3.0).

### Spatial transcriptomics analysis

All spatial transcriptomics data were preprocessed using standard quality control procedures. Raw data were normalized, and low-quality spots were filtered out based on gene count thresholds and mitochondrial gene content. B cell regions were identified using a gene set comprising "CD79A", "CD79B", "BLNK", "CD19", and "MS4A1". UCell^35^ (version 2.4.0) scoring was performed to quantify the expression of these markers, with spots scoring above 0.1 defined as B cell regions. Epithelial regions were classified into RAC1-low and RAC1-high regions based on the mean expression of RAC1. The R package "SPOTlight"^36^ (version 1.4.1) was used to perform deconvolution and annotation of spatial transcriptomics data. SpatialFeaturePlot was used to visualize.

### Prognostic model construction

Pan-cancer data were integrated, and batch effects were removed. The TCGA dataset was split into a training set (60%) and a test set (40%). A multivariate regression analysis was conducted to construct a prognostic model using RAC1 expression and scores for B cells naive, B cells memory, and Plasma cells generated by CIBERSORT (version 0.1.0). The prognosis risk score was defined by a combination of selected predictors weighted by their Cox regression β coefficients. The prognostic model was validated in the test set using survival curves, with the median score as the cutoff. Additionally, the model was validated using three independent datasets [Fudan University Shanghai Cancer Center's (FUSCC) TNBC cohort^37^, METABRIC (https://github.com/cBioPortal/datahub), GSE30219^22^]. In these datasets, survival curves were analyzed using the optimal cutoff value for stratification.

### Cell lines and cultures

The triple-negative breast cancer MDA-MB-231, BT-549 cell lines, non-small cell lung carcinoma A549, NCI-H1395 cell lines and gastric cancer cell line AGS were obtained from American Type Culture Collection (ATCC) (Manassas, VA). MDA-MB-231, BT-549, A549, NCI-H1395 and AGS were cultured in high-glucose Dulbecco's Modified Eagle's Medium (DMEM) (Cytiva, USA) supplemented with 10 % Fetal Bovine Serum (FBS) (FBS, ExCell, China). NCI-H1395 cells were raised in were maintained in RPMI 1640 medium (Gibco, Thermo Fisher, China) supplemented with 10% fetal bovine serum (FBS, ExCell, China). All cells were incubated in a constant-temperature incubator at 37℃ with 5 % CO_2_.

### Knock down the expression of RAC1 in cell lines

MDA-MB-231, BT-549, A549, NCI-H1395 and AGS cell lines were seeded in a 6-well plate to reach 50-70% confluency on the day of transfection. Small interfering RNA (siRNA) and Lipofectamine™ RNAiMAX (Thermo, 13778150) were separately mixed in Opti-MEM. These two components were then combined and incubated at room temperature for 15-20 minutes. The transfection complex was added to the cells, which were incubated for 4-6 hours. After the incubation, the transfection medium was replaced with complete medium, and the cells were cultured for an additional 48-72 hours. The sequences provided for hRAC1 siRNAs are as follows:

NC si:

Sense: UUCUCCGAACGAGUCACGUTT

Antisense: ACGUGACUCGUUCGGAGAATT

hRAC1 si-1:

Sense: AAGGAGAUUGGUGCUGUAAAATT

Antisense: UUUUACAGCACCAAUCUCCUUTT

hRAC1 si-2:

Sense: AGACGGAGCUGUAGGUAAATT

Antisense: UUUACCUACAGCUCCGUCUTT

For RAC1 knockdown using the CRISPR system, lentivirus was produced by co-transfecting HEK293T cells with the desired plasmid, along with psPAX2 and pMD2.G (Addgene). After 72 hours, the virus was harvested by filtering through a 0.45 µm filter. The collected lentivirus was either used immediately to infect target cells with the addition of 8 µg/ml polybrene (H9268, Sigma), or stored at -80°C for future use. Infected cells were then selected with puromycin (ant-pr-1, InvivoGen) at a concentration of 2 µg/ml. RAC1 was knocked out in the A549 cell line using Cas9 sgRNA plasmids (Addgene). The sequences of sgRNAs are follows:

crspRAC1-1-S: CACCGAGTGTGTGGTGGTGGGAGAC

crspRAC1-1-AS: AAACGTCTCCCACCACCACACACTC

crspRAC1-2-S: CACCGCTTCGTCAAACACTGTCTTG

crspRAC1-2-AS: AAACCAAGACAGTGTTTGACGAAGC

### Western blotting

Proteins were extracted using radioimmunoprecipitation assay (RIPA) buffer, supplemented with protease and phosphatase inhibitors. Protein concentrations were determined using a bicinchoninic acid (BCA) quantification kit. The proteins were then separated on 10% acrylamide sodium dodecyl sulfate polyacrylamide gel electrophoresis (SDS-PAGE) gels and subsequently transferred to a polyvinylidene difluoride (PVDF) membrane. Following blocking with 5 % skim milk, the membrane was incubated overnight at 4℃ with primary antibodies. Then, the membrane underwent three 10-min washes with TBST (TBS + Tween), followed by incubation with secondary antibodies at room temperature for 1h. Immunoreactive signals were detected using an enhanced chemiluminescence (ECL) development kit (Merck Millipore, Billerica, MA) and analyzed using Image Lab 6.1 software (Bio-Rad Laboratories, USA). The following antibodies were used: Beta Actin (60008-1-Ig, 1:1000, Proteintech); RAC1 (66122-1-Ig, 1:1000, Proteintech).

### CCK-8 viability assay

A total of 1000-3000 cells per well were initially seeded into 96-well plates. Measurements were started after 24h and extended for seven consecutive days. Cell Counting Kit-8 (CCK8) was combined with serum free medium at 1:10 ratio and introduced into the 96-well plates at, 100μl per well, followed by a 2h incubation at 37℃. Subsequently, the microplate reader was used to quantify absorbance at 450 nm. Technical replicates were performed 3 times for each condition.

### Cloning formation experiment

Following the instructions provided by the transfection reagent kit, Opti-MEM™ (Gibco, Thermo Fisher, China), RAC1- NC/Si, and the transfection agent (Lipo8000, Beyotime, China) were combined and allowed to incubate. The mixture was subsequently distributed evenly across the wells of a 6-well plate, into which cells were then seeded. After the cells had formed colonies, they were fixed using 4% paraformaldehyde and stained with 0.5% crystal violet. The colonies were subsequently imaged and quantified for statistical evaluation.

### Transwell migration assay

For transwell migration assay, a total of 15 × 10^4^ of MDA-MB-231, 15 × 10^4^ BT549, 15 × 10^4^ A549, 15 × 10^4^ NCI-H1395 and 15 × 10^4^ of AGS cells were suspended in 200 μl of DMEM without FBS and seeded on the top chamber of 24-well plate-sized Transwell inserts (Corning Falcon). The lower chambers contained DMEM with 20% FBS. After incubation for 10 h, the inserts were fixed and stained with crystal violet. Cells in the upper chamber were removed with cotton swabs. The average confluence of migrated cells was analyzed by ImageJ according to three random fields captured by 10× microscope. Each experiment was conducted in triplicate.

### Animal experiment

The animal procedures and study designs were approved by the Institutional Animal Care and Use Committee (IACUC) of Fudan University Shanghai Cancer Center (FUSCC) under approval number FUSCC-IACUC-S2023-0421. All protocols adhered to the ethical guidelines set by the IACUC and the institution. Mice were housed in a pathogen-free environment, with controlled room conditions (temperature: 18-23°C, humidity: 40-60%, and a 12-hour light/dark cycle). For each experiment, animals were randomly allocated to the experimental groups, and no blinding was applied. The study used BALB/c nude mice, aged 6-8 weeks. For *in vivo* tumor growth and survival assessment, 6-8-week-old BALB/c nude mice were first anesthetized and their injection sites were shaved. A total of 1×10⁶ A549 cells were suspended in D-PBS (Gibco) and combined in a 1:1 ratio with BME (Cultrex, 3632-010-02), yielding a final concentration of 1×10⁷ cells/ml. The cell suspension was then injected subcutaneously into the dorsal subcutaneous tissue of the mice. Tumor dimensions were measured every 2-3 days using an electronic caliper. Tumor volume was calculated using the formula (length × width² / 2), where length represents the largest tumor diameter and width represents the perpendicular tumor diameter.

### Statistical analysis

In this research, GraphPad Prism 8.3.0 and R software (version 4.3.1) were utilized to process, visualize, and perform statistical evaluations of all data. Thresholds were established for statistical significance as follows: *p* < 0.05 (*), *p* < 0.01 (**), *p* < 0.001 (***), and *p* < 0.0001 (****).

## Results

### The landscape of the expression of RAC1 in pan-cancer levels

**Figure 1:**
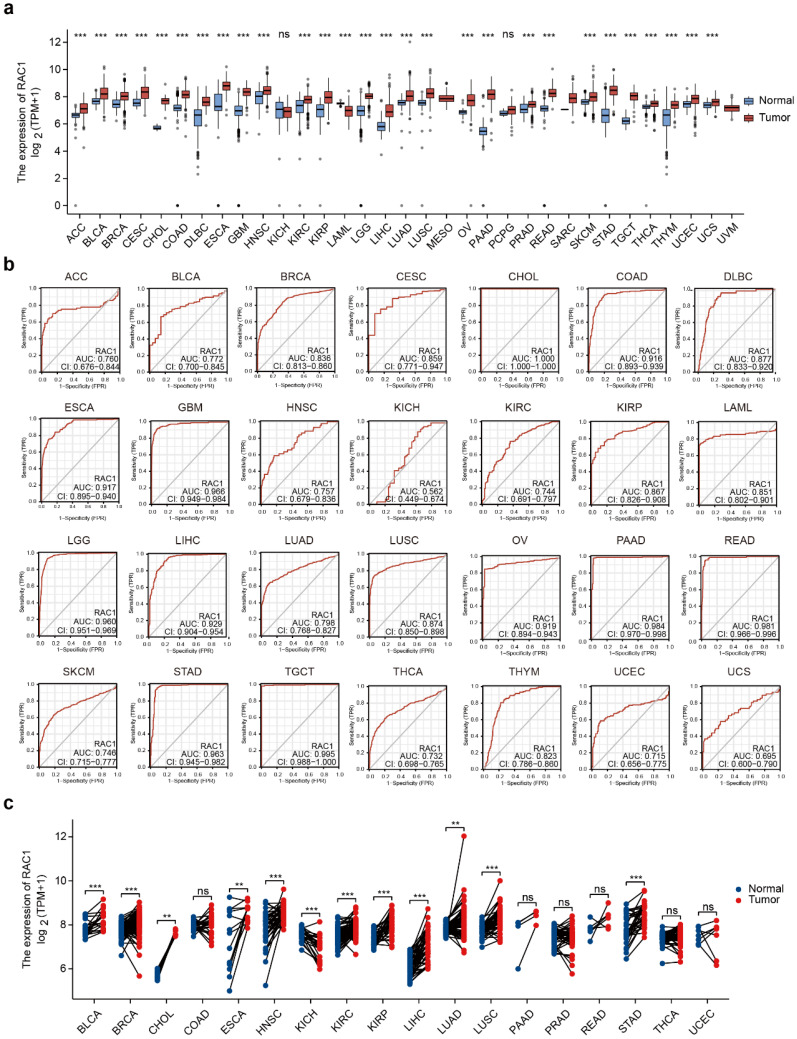
** The landscape of the expression of RAC1 in pan-cancer levels. (a)** Difference expression of RAC1 between normal and tumour tissues across 33 types of cancers in TCGA. (b) ROC curve of RAC1 in 28 types of cancers in TCGA. (c) Differential expression of RAC1 in paired normal and tumour tissues from 18 types of cancers in TCGA. Red indicates tumours, and blue indicates normal. (* p < 0.05, ** p < 0.01, *** p < 0.001, **** p < 0.0001).

To explore RAC1 expression in primary cancers versus adjacent normal tissues, we analyzed RNA sequencing data from TCGA and GTEx. With the exception of cancers without corresponding normal tissues (MESO and UVM), our analysis indicated that RAC1 significantly upregulated in 27 cancers, including ACC, BLCA, BRCA, CESC, CHOL, COAD, DLBC, ESCA, GBM, HNSC, KIRC, KIRP, LGG, LIHC, LUAD, LUSC, OV, PAAD, PRAD, READ, SKCM, STAD, TGCT, THCA, THYM, UCEC, and UCS (Fig. 1a). Among these, the increase in RAC1 expression is most pronounced in CHOL, PAAD, and STAD, highlighting its potential role in gastrointestinal (GI) tumors. However, RAC1 expression is significantly downregulated in LAML.

To investigate whether RAC1 can accurately diagnose tumors, we conducted ROC curve analyses and calculated the AUC values for RAC1 at the pan-cancer level. RAC1 demonstrated strong predictive capacity across 28 types of cancers, with AUC values ranging from 0.695 to 1.00 (Fig. 1b). Tumors with an AUC value above 0.95 include CHOL, GBM, LGG, PAAD, READ, STAD, and TGCT. The ROC analysis revealed that RAC1 serves as a reliable diagnostic marker.

We further analyzed the differences in RAC1 expression levels between paired tumor and normal tissues (Fig. 1c). RAC1 expression remains significantly elevated in the paired tissues of BLCA, BRCA, CHOL, ESCA, HNSC, KIRC, KIRP, LIHC, LUAD, LUSC, and STAD, suggesting that RAC1 plays an even more critical role in these tumors. In the paired tissues of KICH, RAC1 mRNA expression is significantly lower. There is no statistically significant difference in RAC1 expression in PAAD.

### High RAC1 expression is associated with advancing stages and poor prognosis

**Figure 2:**
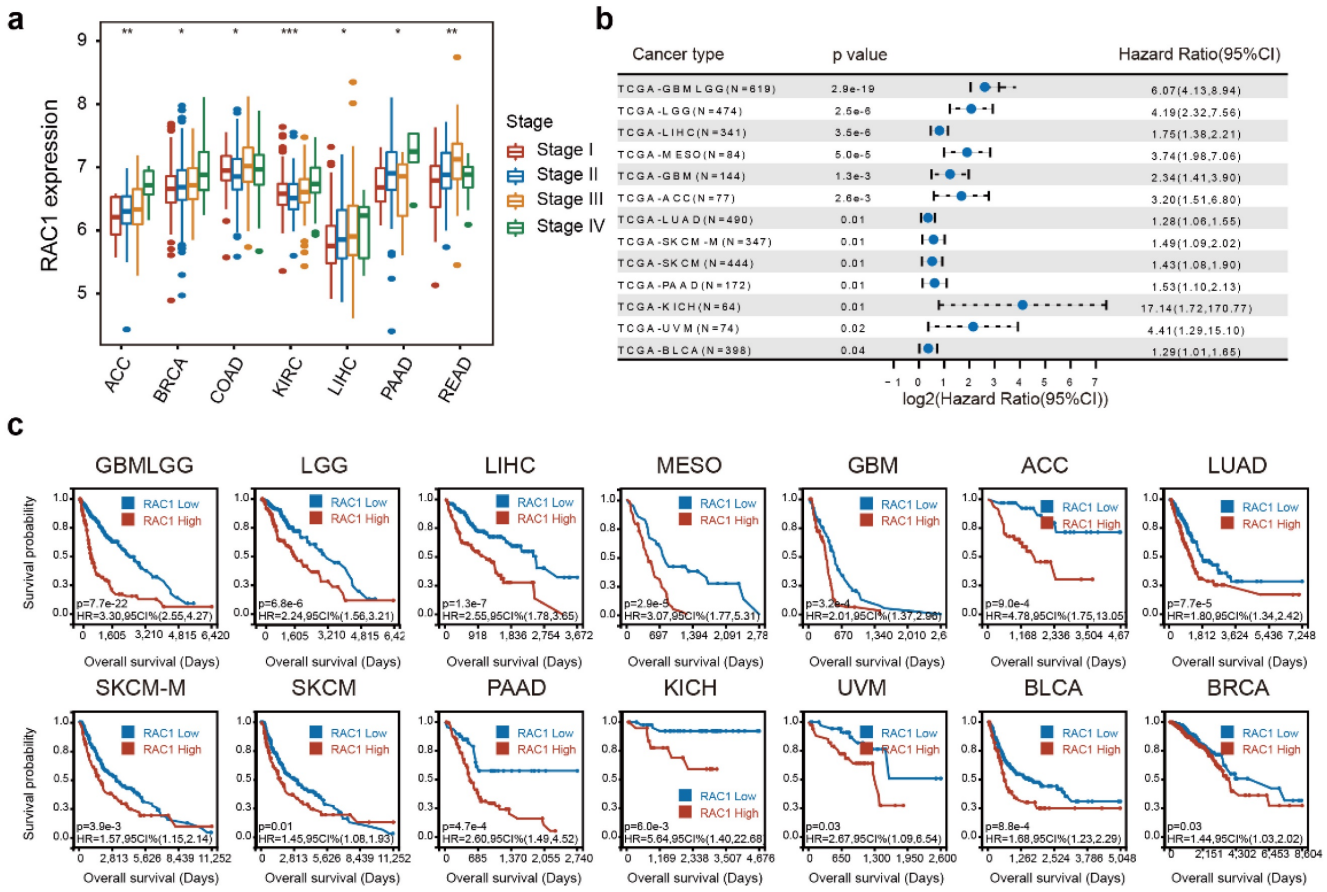
** RAC1 is associated with higher clinical stages and poorer OS in various cancers.** (a) Forest plot about the results of survival analysis of RAC1 expression on OS in 13 types of cancer with a Pvalue < 0.05. (b) Boxplot shows the correlation between RAC1 and clinical stages in 7 types of cancer. (c) Kaplan-Meier plot of significance between RAC1 expression and OS ((P < 0.05)). Red indicates high group, and blue indicates low group.

The integrated analysis of RAC1 expression across different clinical stages (I, II, III, and IV) at the pan-cancer level reveals a positive correlation between RAC1 expression and clinical stages in 7 cancers, including ACC, BRCA, COAD, KIRC, LIHC, PAAD, and READ (Fig. 2a). Notably, RAC1 expression progressively increases with advancing clinical stage in ACC, BRCA, KIRC, LIHC, and PAAD, suggesting that RAC1 is associated with tumor progression in these cancers.

An integrated analysis of the three indicators revealed that higher expression of RAC1 is associated with poorer clinical prognosis, identifying RAC1 as a hazard factor for many cancers. For OS, RAC1 was found to be a hazard factor for GBMLGG, LGG, LIHC, MESO, GBM, ACC, LUAD, SKCM, PAAD, KICH, UVM, and BLCA, with a p-value < 0.05 (Fig. 2b). Notably, the hazard ratio in KICH was as high as 17.14 (P = 0.01). Kaplan-Meier survival analysis of OS data demonstrated a significant correlation between RAC1 expression and unfavorable prognosis in GBMLGG, LGG, LIHC, MESO, GBM, ACC, LUAD, SKCM, PAAD, KICH, UVM, BLCA, and BRCA (Fig. 2c). Additionally, univariate Cox regression analysis of DSS and PFI data at the pan-cancer level was conducted to further evaluate the prognostic value of RAC1. The analysis demonstrated that higher RAC1 expression is positively correlated with poor prognosis in GBMLGG, LGG, MESO, GBM, ACC, LIHC, KICH, UVM, LUAD, PAAD, CESC, and SKCM concerning DSS (Fig. S1a), and in GBMLGG, LGG, ACC, MESO, UVM, PAAD, KIPAN, and STES regarding PFI (Fig. S1b). In conclusion, RAC1 may serve as a significant clinical predictor for the tumors mentioned above.

### Knockdown of RAC1 inhibits the proliferation and migration of breast cancer and lung cancer cells

**Figure 3:**
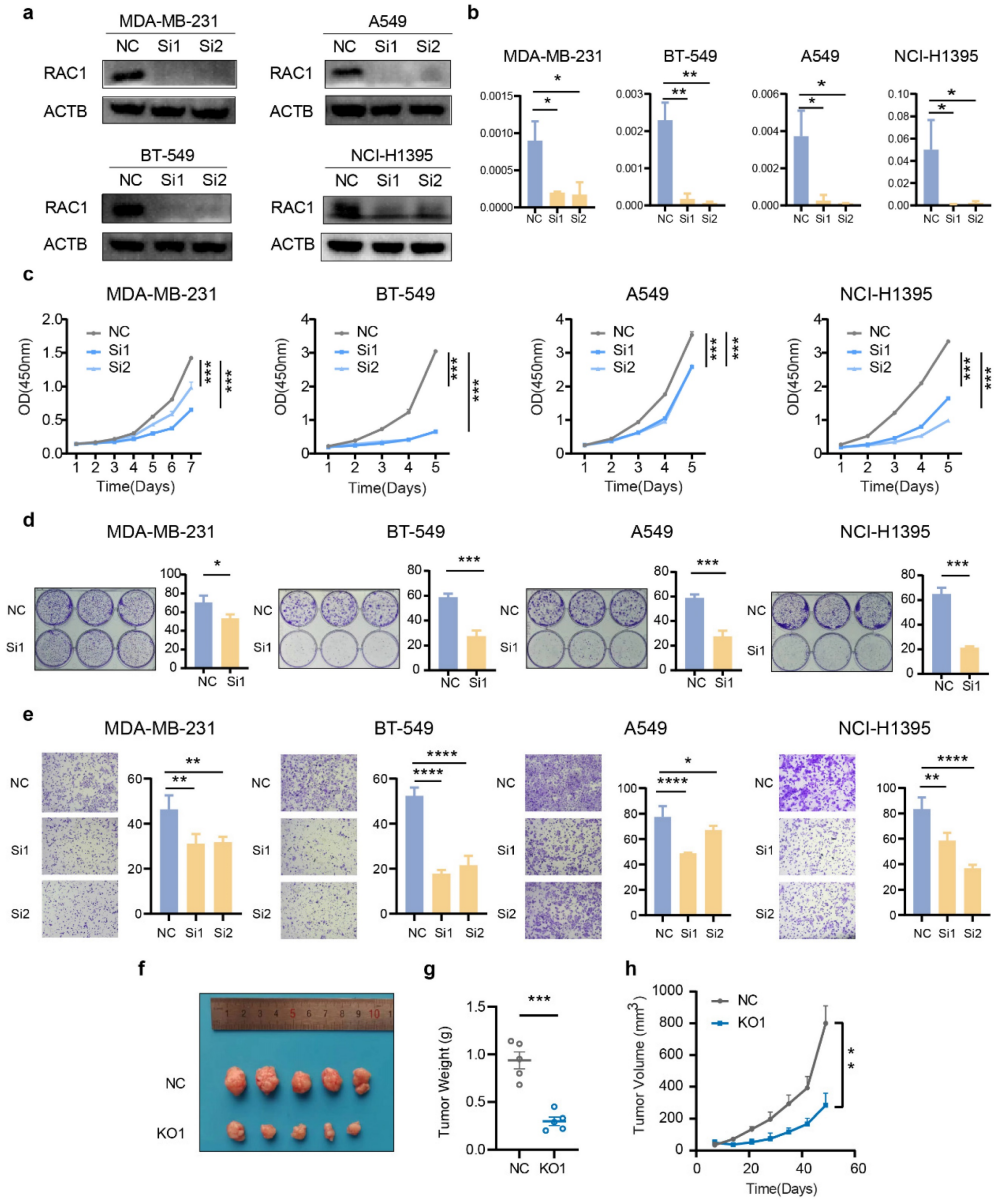
** Knock down RAC1 in breast cancer and lung cancer cells suppress cell proliferation and migration.** (a-b) The knockdown efficiency of RAC1 in MDA-MB-231, BT549, A549, and NCI-H1395 cells at the protein level and RNA level. (c) The CCK-8 proliferation assay indicates that knockdown of RAC1 can significantly reduce the proliferation efficiency of MDA-MB-231, BT549, A549, and NCI-H1395 cell lines. (d) The colony formation assay indicates that RAC1 knockdown significantly reduces colony formation. Representative images of colony formation assays (left) and quantification of the colony numbers (right) are shown. (e) The transwell assay indicates that RAC1 knockdown significantly reduces the migratory ability of these cell lines. Representative images of Transwell migration assays (left) and quantification of the migrated cells (right) are shown. (f-h) *In vivo* experiments demonstrated that knockdown of RAC1 in nude mice reduces the proliferation efficiency of A549 cells. NC represents the negative control, Si1 and Si2 represent two different RAC1 knockdown groups. (* p < 0.05, ** p < 0.01, *** p < 0.001, **** p < 0.0001).

To validate the pivotal role of RAC1 in tumor development, we performed functional assays using five cell lines representing three cancer types: breast cancer (MDA-MB-231 and BT549), lung adenocarcinoma (A549 and NCI-H1395), and gastric cancer (AGS). First, we constructed knockdown cell lines for these four cell types using siRNA. Western blot and qPCR validated the knockdown efficiency of RAC1 (Fig. 3a-b, Fig. S2a-b). The CCK-8 proliferation assay indicated that knockdown of RAC1 significantly reduced the proliferative capacity (Fig. 3c, Fig. S2c). Cloning formation experiments demonstrated a significant reduction in the proliferation capacity when knock downing RAC1 (Fig. 3d, Fig. S2d). The Transwell migration assay demonstrated the migration-promoting function of RAC1 in these cell lines, with RAC1 knockdown significantly reducing the migratory ability (Fig. 3e, Fig. S2e). Finally, tumor formation assay in nude mice confirmed that knockdown of RAC1 can reduce the proliferation of A549 cells *in vivo* (Fig. 3f-h). In summary, these observations provided persuasive evidence at the biological level to support the oncogenic role of RAC1 in breast cancer and lung adenocarcinoma.

### Correlation between expression of RAC1 and CNV status, TMB and MSI

To explore the correlation between RAC1 expression and genomic alterations, we examined data on RAC1 expression and CNV status across 33 types of cancers. We observed that an elevated expression of RAC1 is associated with a Gain status in CNV, surpassing the expression observed in instances where CNV status was either Neutral or characterized by a Loss in 20 tumour types, including GBM, GBMLGG, LGG, CESC, LUAD, COAD, READ, BRCA, ESCA, STES, SARC, STAD, UCEC, HNSC, LUSC, LIHC, MESO, OV, TGCT, SKCM and BLCA (Fig. S3a). At the genomic level, CNV status may be a key determinant affecting the expression of RAC1.

The TMB and MSI were considered as potential biomarkers for predicting response to immunotherapy and clinical outcomes. The radar chart indicates a positive correlation between RAC1 expression and TMB in BLCA, ESCA, UCS, THYM, STAD, PAAD, MESO and LGG, while in COAD and OV, a negative correlation is observed (Fig. S3b). Regarding MSI, RAC1 expression shows a positive correlation in CHOL, DLBC, LIHC, PCPG, READ, and STAD. Conversely, in CESC, LGG, and PRAD, the correlation with RAC1 expression is negative (Fig. S3c). The results revealed a broad distribution of RAC1 expression in cancers characterized by either elevated or reduced TMB and MSI.

### High RAC1 expression is associated with increased immune checkpoint activities and low stromal and immune scores in various cancers

To more specifically investigate the impact of RAC1 on the immune microenvironment, we analyzed the relationship between RAC1 expression and the activity of immune checkpoints and immunosuppressive factors at the pan-cancer level. The results depicted that RAC1 expression was positively correlated with many immune checkpoints, such as PDCD1, LAG3, CTLA4, HAVCR2, and TIGIT, and immunosuppressive factors, such as CSF1R, IL10, TGFB1, TGFBR1, and VTCN1, in most cancer types, namely ACC, BLCA, BRCA, KICH, KIRC, KIRP, LGG, LIHC, PAAD, PCPG, PRAD, and UVM. However, in ESCA and LUSC, the condition was the opposite (Fig. S4a, Fig. S4b).

The ESTIMATE algorithm was utilized to calculate the StromalScore, ImmuneScore, and ESTIMATEScore for RAC1 across 33 types of cancers. The analysis revealed that RAC1 expression was significantly negatively correlated with StromalScore, ImmuneScore, and ESTIMATEScore in ESCA, HNSC, LUSC, SKCM, STAD, TGCT, and UCEC (Fig. S4c). In COAD, THCA, and THYM, RAC1 expression was negatively correlated with ImmuneScore and ESTIMATEScore. Conversely, a positive correlation was observed in KIRC, LAML, LGG, LIHC, and PCPG. Overall, these findings suggest that high RAC1 expression is generally associated with lower stromal and immune scores in the tumor microenvironment of most cancers.

### Potential influence of RAC1 on B lineage cell infiltration in the tumor microenvironment

**Figure 4:**
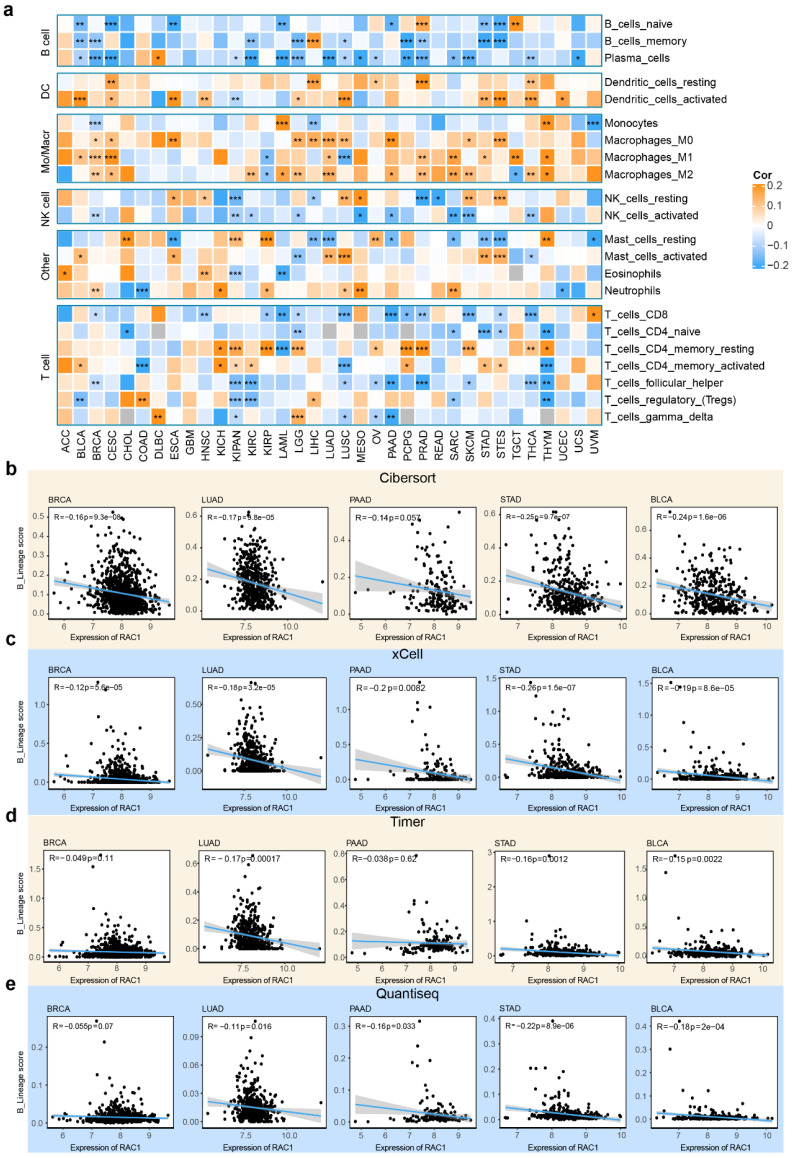
** RAC1 is negatively correlated with B infiltration. (a)** The heatmap displays the results of the CIBERSORT analysis of RAC1 at the pan-cancer level.(b-e) The correlation dot plot illustrates the relationship between RAC1 and B cell infiltration using the CIBERSORT, Xcell, Timer and Quantiseq in BRCA, LUAD, PAAD, STAD and BLCA. (* p < 0.05, ** p < 0.01, *** p < 0.001, **** p < 0.0001).

The tumor immune microenvironment is composed of various subsets of immune cells, each contributing to distinct immunological niches. To investigate the relationship between RAC1 expression and immune cell infiltration, we employed the CIBERSORT algorithm to analyze 22 types of immune cell infiltration scores within the tumor microenvironment. Interestingly, our analysis revealed a significant negative correlation between RAC1 expression and B cell lineage infiltration scores across most cancers. Specifically, RAC1 is negatively correlated with all three B cell subsets—naïve B cells, memory B cells, and plasma cells—in cancers such as BLCA, BRCA, CESC, KIRC, LAML, LGG, STAD, and STES (Fig. 4a).

To further investigate the potential relationship between RAC1 and B cell infiltration, we employed xCell, TIMER, and Quantiseq to calculate infiltration scores of immune subsets in the tumor microenvironment. Integrating infiltration scores from these algorithms allowed us to derive an overall B cell infiltration score, termed B_lineage scores. We observed that in the CIBERSORT algorithm, RAC1 was negatively correlated with B_lineage scores in BRCA (R = -0.16, P = 9.3e-08), LUAD (R = -0.17, P = 9.8e-05), PAAD (R = -0.14, P = 0.057), STAD (R = -0.25, P = 9.7e-07), and BLCA (R = -0.24, P = 1.6e-06) (Fig. 4b). Similar negative correlations were observed in the xCell algorithm for LUAD (R = -0.18, P = 3.2e-05), PAAD (R = -0.2, P = 0.0082), STAD (R = -0.26, P = 1.5e-07), and BLCA (R = -0.19, P = 8.6e-05) (Fig. 4c). Using the TIMER algorithm, this phenomenon was also noted in LUAD (R = -0.17, P = 0.00017), STAD (R = -0.16, P = 0.0012), and BLCA (R = -0.15, P = 0.0022) (Fig. 4d). Finally, the Quantiseq algorithm confirmed negative correlations in LUAD (R = -0.11, P = 0.016), PAAD (R = -0.16, P = 0.033), STAD (R = -0.22, P = 8.9e-06), and BLCA (R = -0.18, P = 2e-04) (Fig. 4e). Notably, STAD and BLCA consistently showed significant negative correlations between RAC1 expression and B cell infiltration across all four algorithms, suggesting a potential immunosuppressive role of RAC1 in these tumors. These findings suggest that high RAC1 expression may be associated with reduced B cell infiltration, indicating a potential influence of RAC1 on the immune microenvironment in various cancers.

**Figure 5:**
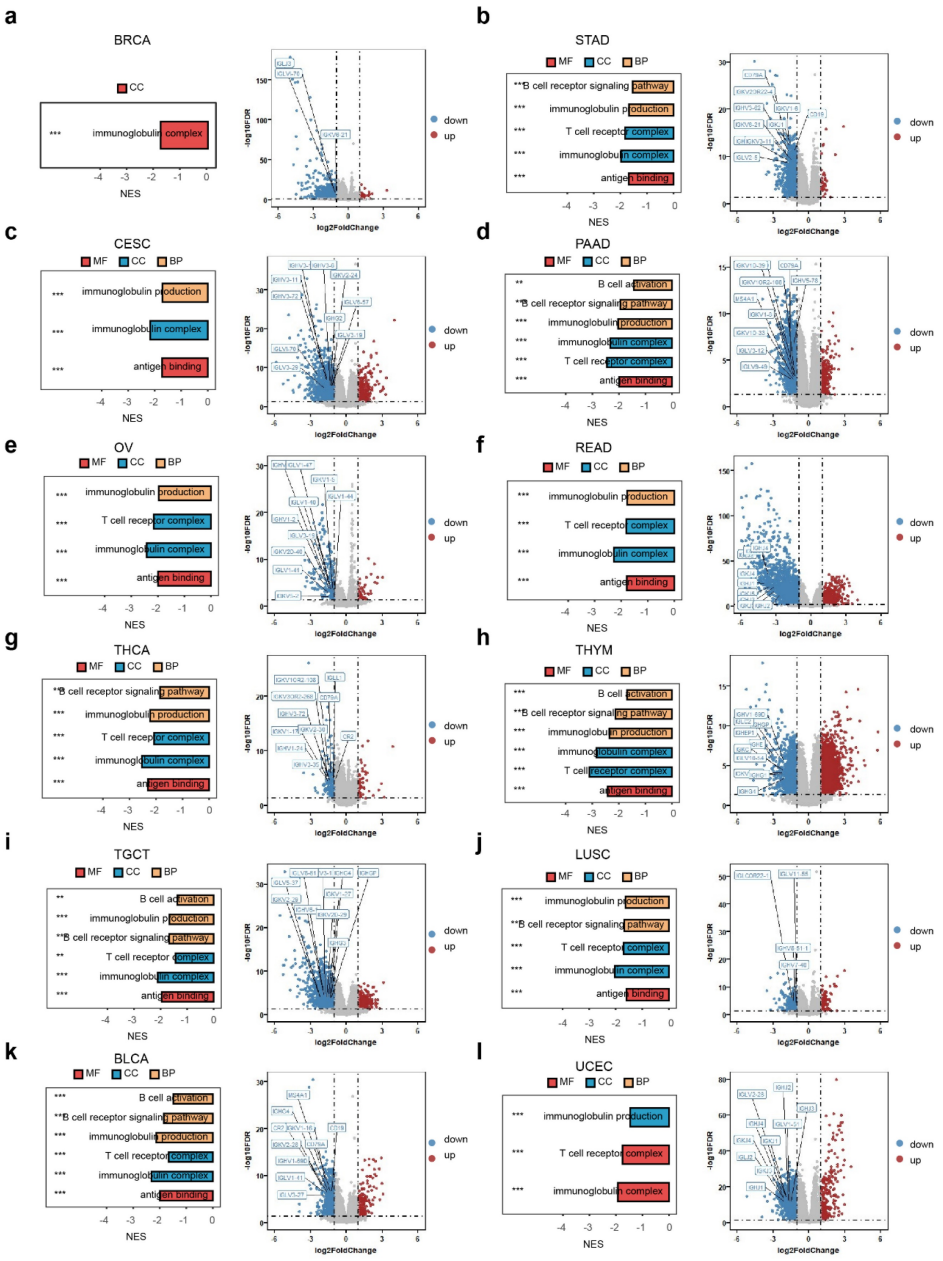
** The GSEA enrichment analysis and differential gene analysis of RAC1 in 12 types of cancers are related to the B cell activation pathway and B cell related genes. (a-l)** The bar chart and volcano plot respectively display the B cell activation pathways associated with RAC1 and the top 10 B cell-related differentially expressed genes ranked by FoldChange in 12 type of cancers. (* p < 0.05, ** p < 0.01, *** p < 0.001, **** p < 0.0001).

Based on the aforementioned research, we utilized differential expressed genes for GSEA analysis to explore the biological roles of RAC1 within the immune microenvironment across different cancer types. Our GSEA enrichment analysis at the pan-cancer level revealed that B cell-related pathways are predominantly enriched in the downregulated pathways in 12 types of cancer, including BRCA, STAD, CESE, PAAD, OV, READ, THCA, THYM, TGCT, LUSC, UCEC, and BLCA (Fig. 5a-l).

Additionally, volcano plots of the differentially expressed genes in these cancers indicated that in the RAC1 low-expression group, the expression levels of B cell surface markers (such as CD19, CD79A, and MS4A1) and immunoglobulin-related genes (such as IGHG1 and CR2) were higher. These observations highlight a potential inverse relationship between RAC1 expression and B cell infiltration. Taken together, these results suggest that RAC1 may influence the immune microenvironment and potentially affect the activation and maturation of B cells.

### Pan-cancer single cell and spatial transcriptomics reveals RAC1's role in tumor immune microenvironment and B cell infiltration

The composition of the tumor microenvironment is intricate, and single-cell level analysis is essential for understanding the occurrence and development of tumors. To precisely investigate the primary cell types through which RAC1 exerts its effects in the immune microenvironment, we utilized the TISCH2 web tool to analyze RAC1 expression in different microenvironmental cell subpopulations. This single-cell analysis, spanning 14 types of cancers, revealed that RAC1 is expressed in a variety of cell subpopulations, including CD8 T cells, dendritic cells (DC), endothelial cells, and fibroblasts. Notably, RAC1 was predominantly expressed in epithelial cells, malignant cells, and monocytes/macrophages (Mono/Macro cells) across these cancer types (Fig. S5).

**Figure 6:**
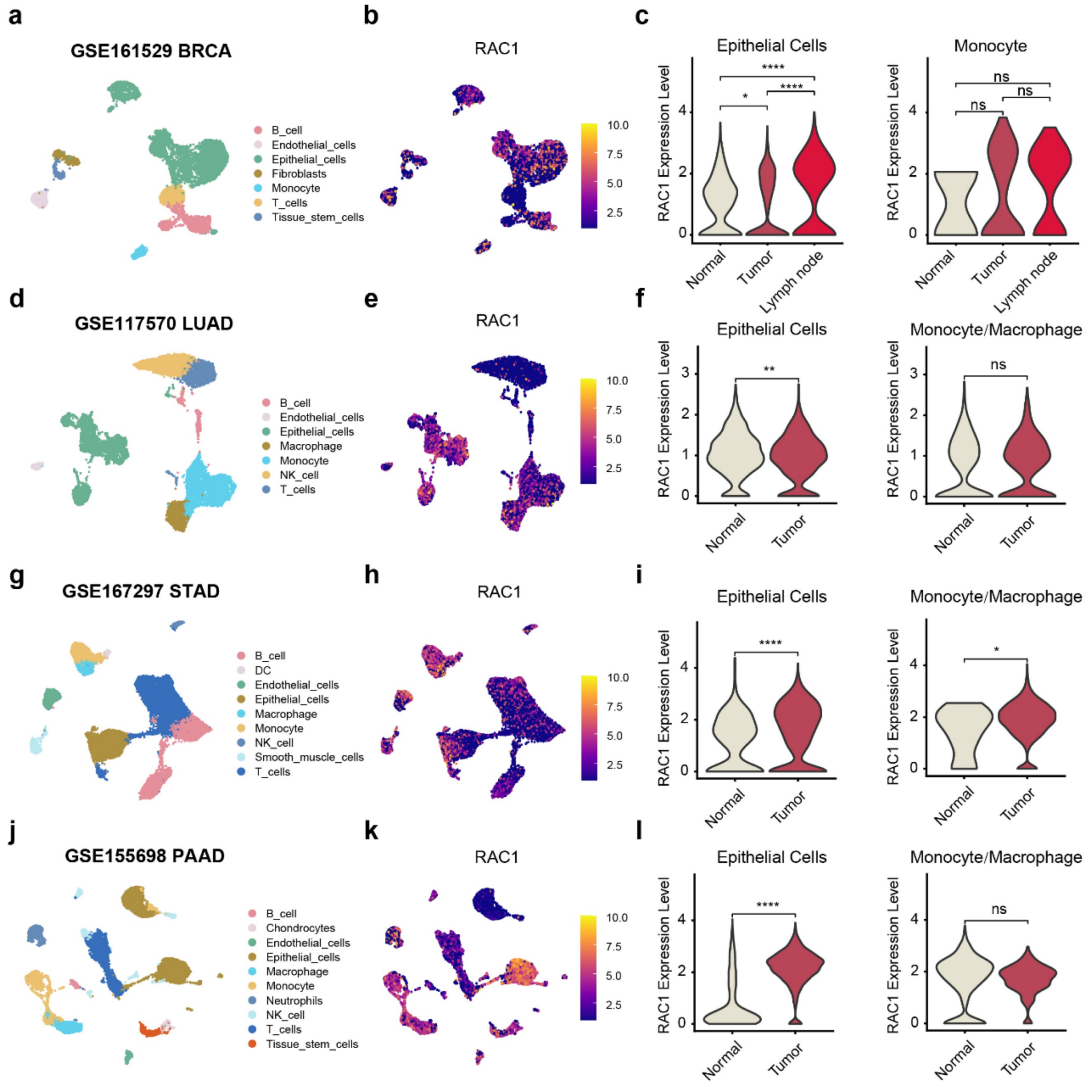
** RAC1 primarily exerts its main biological functions in epithelial cells.** UMAP plot visualization of the cell subpopulations of the tuomr microenvironment in (a)BRCA, (d)LUAD, (g)STAD and (j)PAAD. The Featureplot illustrates the expression distribution of RAC1 across various cell subsets in (b)BRCA, (e)LUAD, (h)STAD and (j)PAAD. The violin plot displays the expression differences of RAC1 in epithelial cells and Mono/Macro cells between paired normal, tumour tissues and metastatic lymph nodes in (c)BRCA, (f)LUAD, (i)STAD and (l)PAAD. (* p < 0.05, ** p < 0.01, *** p < 0.001, **** p < 0.0001).

To explore the role of RAC1 in the immune microenvironment, we collected single-cell sequencing data from paired normal and tumor tissues for BRCA^14^, LUAD^15^, PAAD^17^ and STAD^16^ from public databases, with BRCA also including paired metastatic lymph nodes. The markers used to annotate cell subtypes are shown in (Fig. S6a-d). In the single-cell data for BRCA, RAC1 expression showed a significant progressive increase in epithelial cells from normal tissue to tumor, and further to metastatic lymph nodes, while no significant difference in expression was observed in monocyte cells (Fig. 6a-c). In the single-cell data of LUAD and PAAD, the expression of RAC1 in tumour epithelial cells is significantly higher than that in normal epithelial cells. And there is no significant difference in the expression of RAC1 on Mono/Macro cells between tumour and normal tissues (Fig. 6d-f, j-l). In the STAD single-cell data, although the expression of RAC1 on Mono/Macro cells in tumour tissue has increased compared to normal tissue, it can be observed that the increases is not as significant as the rise in epithelial cells (Fig. 6g-i). We hypothesize that the high expression of RAC1 contributes significantly to the progression of epithelial malignancies.

**Figure 7:**
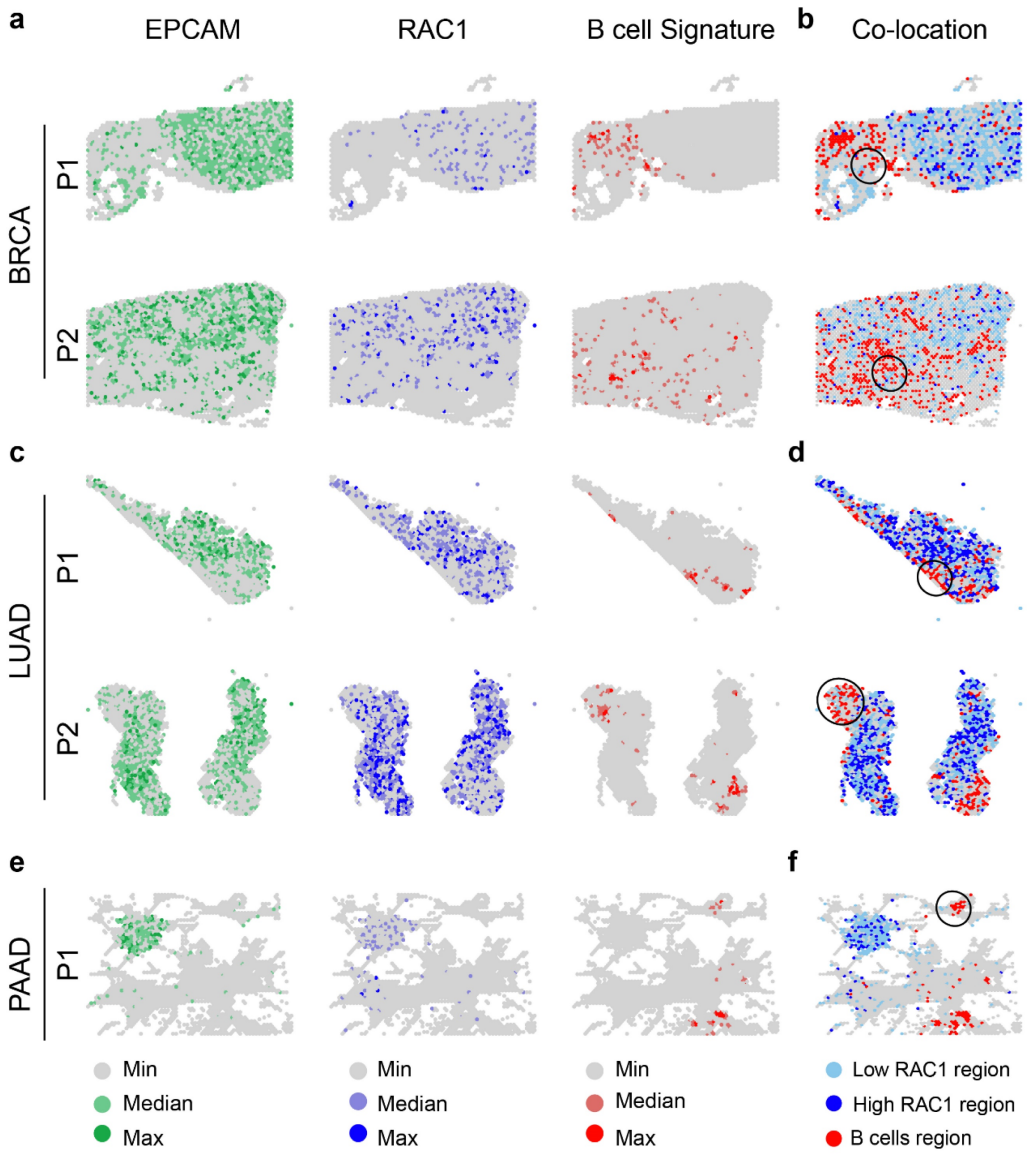
** Spatial transcriptomics analysis reveals the spatial localization relationship between B cells and RAC1 epithelial cells.** (a) Spatial maps of BRCA samples (P1 and P2) displaying the distribution of EPACM (green), RAC1 (blue), and B cell signatures (red). (c) Same spatial maps of LUAD samples (P1 and P2). (e) Same spatial maps of PAAD samples. The co-localization panel shows regions where B cells and low RAC1 expressed epithelial cells overlap (highlighted with black circles) in BRCA (b), LUAD (d) and PAAD (f).

To further investigate the hypothesis that there is a negative correlation between RAC1 expression and B cell infiltration, we utilized spatial transcriptomics data^38^ from BRCA^19^, LUAD^20^, and PAAD^21^ to explore the co-localization patterns of B cell regions and RAC1 epithelial regions. We first used SPOTlight to perform deconvolution and annotation of spatial transcriptomics data (Fig. S7a-c), providing a reference for the immune microenvironment. By analyzing the spatial distribution of EPCAM expression, RAC1 expression, and B cell regions, we found that RAC1 is strongly co-localized with EPCAM and B cells are mainly distributed in non-tumor core regions and tumor margin areas (Fig. 7a, c, e). Next, we explored the co-localization relationship between B cell regions and RAC1 epithelial regions. We found that areas surrounding Low RAC1 regions exhibited greater B cell immune infiltration, while High RAC1 regions had significantly fewer B cells (typical regions are circled in black). This trend was consistently observed across all three cancer types (Fig. 7b, d, f). In summary, spatial transcriptomics results indicate that B cells and RAC1 exhibit a mutually exclusive spatial localization relationship, suggesting that RAC1 may impair B cell infiltration in the tumor microenvironment.

### Prognostic signature based on RAC1 expression and B cell infiltration in the pan-cancer levels

Given that our study suggests a potential negative correlation between RAC1 expression levels and B cell infiltration in the tumor microenvironment, it is hypothesized that jointly evaluating RAC1 gene expression and B cell infiltration may offer valuable insights for predicting patient prognosis. Therefore, a prognostic model based on RAC1 gene expression levels and B cell infiltration has been developed.

**Figure 8:**
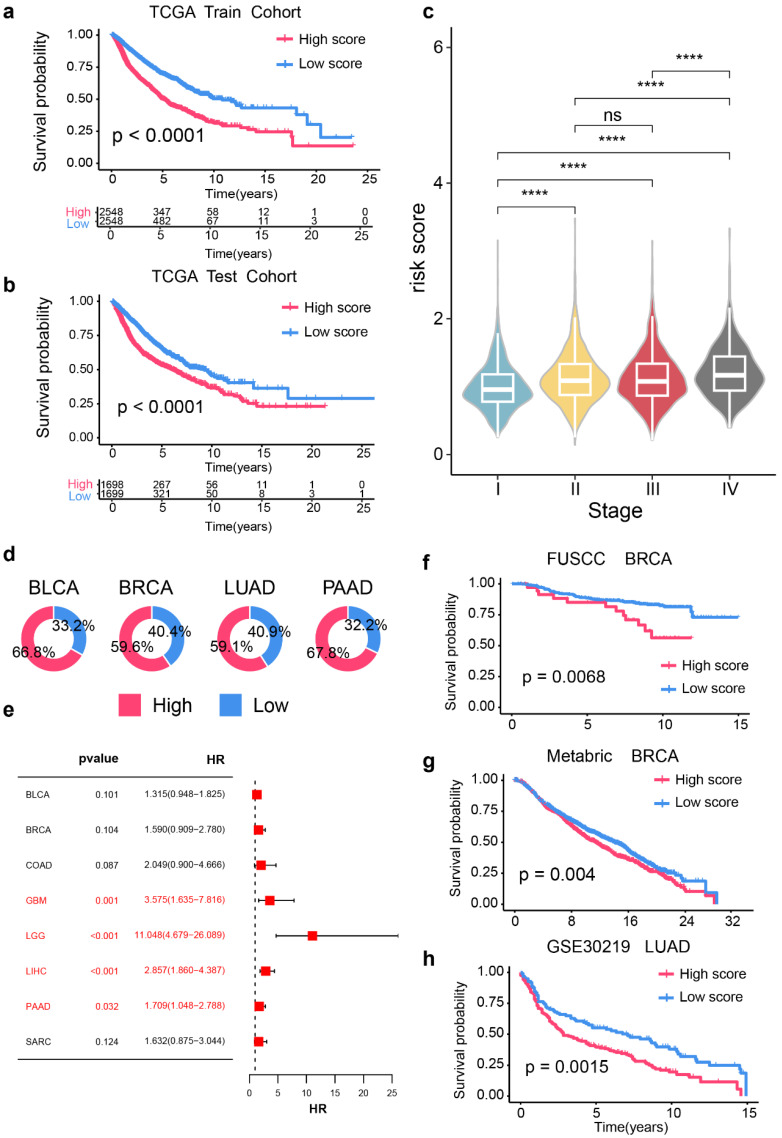
** Construction of prognostic signature based on RAC1 expression and B cell infraction in pan-cancer cohorts.** (a-b) Kaplan-Meier survival curves comparing over survival between high-risk and low-risk score groups in the TCGA training cohort (a) and TCGA test cohort (b). Patients with high-risk scores show significantly poorer survival rates (p < 0.0001). (c) Violin plots depicting the distribution of risk scores across different cancer stages (I-IV). Higher stages are associated with higher risk scores (* p < 0.05, ** p < 0.01, *** p < 0.001, **** p < 0.0001; ns: not significant). (d) Pie charts indicating the proportion of high-risk (red) and low-risk (blue) patients in BLCA, BRCA, LUAD, and PAAD cohorts. (e) Forest plot of hazard ratios (HR) and p-values for the prognostic signature across various cancer types, highlighting significant associations in GBM, LGG, LIHC, and PAAD (p < 0.05). (f-h) Kaplan-Meier survival curves for independent validation cohorts: FUSCC BRCA (f), Metabric BRCA (g), and GSE30219 LUAD (h). Patients with high-risk scores consistently show poorer survival outcomes (FUSCC BRCA: p = 0.0068; Metabric BRCA: p = 0.004; GSE30219 LUAD: p = 0.0015).

First, we partitioned the pan-cancer TCGA dataset into training and test cohorts with a 60% threshold. Next, we selected RAC1 expression levels and CIBERSORT-derived infiltration scores for naïve B cells, memory B cells, and plasma cells to construct the prognostic model. Using multivariate regression analysis of these four parameters, we formulated the RPBI (RAC1 plus B cell Infiltration) score as follows: RPBI score = (0.52920 * RAC1) + (1.67389 * memory B cells score) + (-0.55879 * naive B cells score) + (-0.13764 * plasma cells score). The RPBI score was validated to have a significant predictive effect on cancer prognosis in both the training cohort (Fig. 8a; p < 0.0001) and the test cohort (Fig. 8b; p < 0.0001). Additionally, when the relationship between RPBI and tumor staging was examined, it was found that the RPBI score significantly increased with advancing tumor stages (Fig. 8c). The pie charts illustrate the distribution of high and low groups based on the RPBI score in four types of cancer: BLCA, BRCA, LUAD, and PAAD (Fig. 8d). High RPBI scores are associated with a significantly increased risk of death in in several cancer types, particularly in GBM (HR = 3.575, 95% CI: 1.635 - 7.816, p = 0.001), LGG (HR = 11.048, 95% CI: 4.679 - 26.089, p < 0.001), LIHC (HR = 2.857, 95% CI: 1.860 - 4.387, p < 0.001), and PAAD (HR = 1.709, 95% CI: 1.048 - 2.788, p = 0.032) (Fig. 8e). Finally, to validate the robustness of the RPBI score as a prognostic model, three independent cohorts were used to assess its predictive efficacy. The RPBI score demonstrated significant prognostic performance in the FUSCC TNBC cohort (p = 0.0068), METABRIC (p = 0.004), and GSE30219^22^(p = 0.0015) (Fig. 8f-h). Overall, the prognostic model based on RAC1 and B cell infiltration demonstrates significant prognostic value at the pan-cancer level, indicating that patients with a high RPBI score have poorer prognosis.

## Discussion

This study conducted a comprehensive analysis of the expression patterns of RAC1 at the pan-cancer level and its relationship with cancer progression, prognosis, immune microenvironment. Our findings echo previous studies that have revealed the important role of RAC1 in various cancers^5^,^39^-^41^. Furthermore, our research enhances the understanding of RAC1 as a prognostic biomarker and a driver of tumor progression and malignancy. Genomic alterations are a hallmark of tumor evolution^42^. In this study, we found that CNV amplification significantly increases RAC1 expression, suggesting its potential role as a driver gene in tumor progression and malignant phenotypes. This provides a foundation for investigating its functional mechanisms in promoting cell proliferation, migration, and invasion. Additionally, within the immune microenvironment, our findings suggest that elevated RAC1 expression may modulate B cell infiltration. Spatial transcriptomics analysis suggests that RAC1 expression regions exhibit a certain mutually exclusive co-localization with B cell infiltration regions. Then, we constructed a prognostic model incorporating both RAC1 expression and B cell infiltration. The validity of this model was verified at the pan-cancer level.

While cancer therapies like PD-L1 and CTLA-4 inhibitors have advanced immunotherapy, their effectiveness remains limited^43^,^44^. Few studies have explored RAC1's role in the tumor immune microenvironment, although some indicate RAC1 mutations can upregulate the PD-L1 expression in melanoma^12^ and regulate the function of Th17 cells in autoimmune diseases^45^. Our study identified a significant negative correlation between RAC1 expression and B cell infiltration across cancer types, suggesting that RAC1 may act as a regulator of B cell activity. This is important, as B cells contribute to anti-tumor immunity through antibody production, antigen presentation, and cytokine release^46^-^48^. Studies have shown that higher B cell infiltration^49^ and the existence of B cell-enriched tertiary lymphoid structures^50^, serve as harbingers of favorable outcomes in cancer paitents. Through the application of multiple immune infiltration algorithms, we observed a significant negative correlation between RAC1 expression and B lymphocyte score across various cancer types. Furthermore, spatial transcriptomics data from BRCA, LUAD, and PAAD indicated a mutually exclusive co-localization between regions of high RAC1 epithelial expression and areas of B cell infiltration. These findings suggest that RAC1 may serve as a key regulatory factor influencing B cell infiltration within the tumor microenvironment.

Then, we constructed the RPBI prognostic model based on RAC1 and immune scores of three B cell subtypes (B cells naïve score, B cells memory score, and Plasma cells score) calculated by CIBERSORT. The RPBI risk score demonstrated significant predictive performance in the TCGA training and test cohorts, as well as in independent validation cohorts of BRCA and LUAD. Thus, as a multifaceted prognostic model, the RPBI score may reflect the immune status of tumors and provide comprehensive and reliable prognostic information, potentially offering new perspectives for cancer treatment.

However, this study has limitations. One limitation of this study is its primary reliance on bioinformatics analysis, resulting in a lack of exploration and experimental validation regarding the potential interactions between RAC1 and B cells. The study does not thoroughly investigate the relationship between RAC1 and B cell immune subsets, thereby overlooking the complexity and dual nature of these subsets—where different B cell subsets can exhibit both pro-tumor and anti-tumor functions^51^. This aspect is an important avenue for further in-depth investigation. Moreover, the RPBI model's clinical applicability requires further validation with independent cohorts and deeper mechanistic investigations.

## Conclusion

This study comprehensively analyzed RAC1 expression across various cancers, revealing significant upregulation in many cancer types. RAC1 demonstrated strong diagnostic potential and was associated with advancing clinical stages and poor prognosis. RAC1 expression was linked to genomic alterations, immune checkpoint activity, and reduced B cell infiltration, suggesting its role in shaping the tumor microenvironment. Spatial transcriptomics confirmed a mutually exclusive localization between RAC1 and B cell regions. We developed the RPBI (RAC1 plus B cell Infiltration) prognostic model, which showed strong predictive efficacy across multiple cohorts. Functional experiments supported the oncogenic role of RAC1, highlighting its potential as a prognostic biomarker and therapeutic target.

## Supplementary Material

Supplementary figures.
