# Supplementary material for: Comprehensive Pan-Cancer Analysis of RAC1 Decoding the Impact on Cancer Prognosis and B cell immune Regulation
Source: Int J Med Sci. 2025 Jan 13;22(3):662–79. doi: 10.7150/ijms.104488 (PMC11783080; doi:10.7150/ijms.104488)
Supplement: Supplementary file 1 — Supplementary figures. [file ijmsv22p0662s1.pdf]

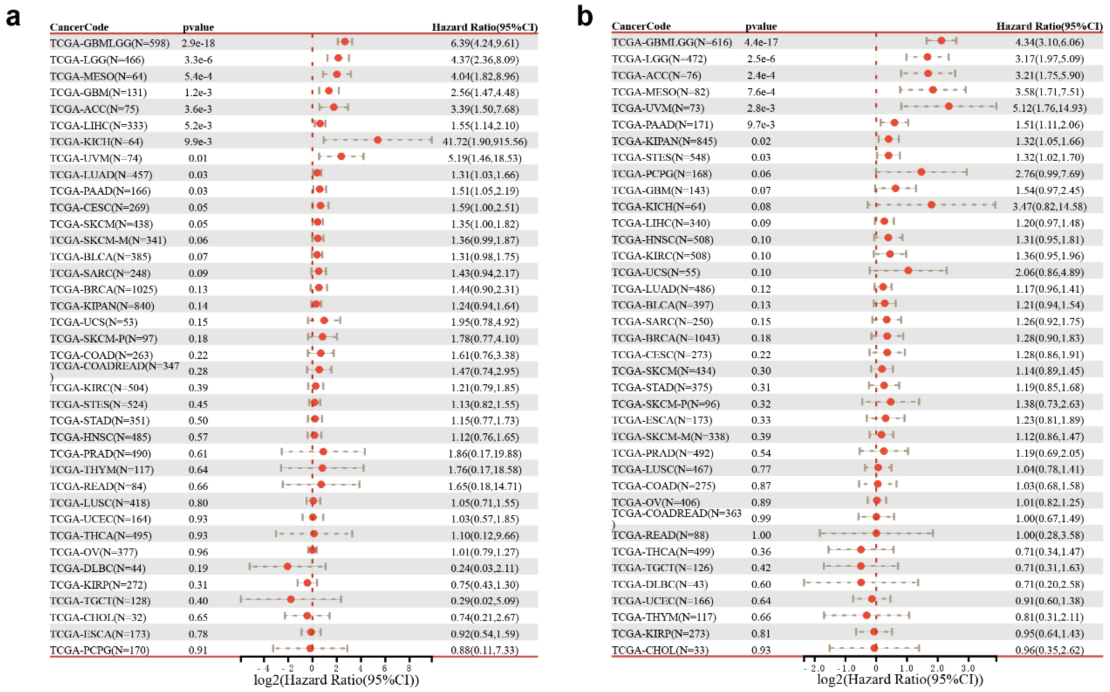

Figure S1 RAC1 expression is significantly correlated with an unfavorable prognosis of DSS and PFI in various cancers. Forest plot depicting the results of survival analysis for RAC1 on (a)DSS and (b)PFI in pan-cancer. (* p < 0.05, ** p < 0.01, *** p < 0.001, **** p < 0.0001).

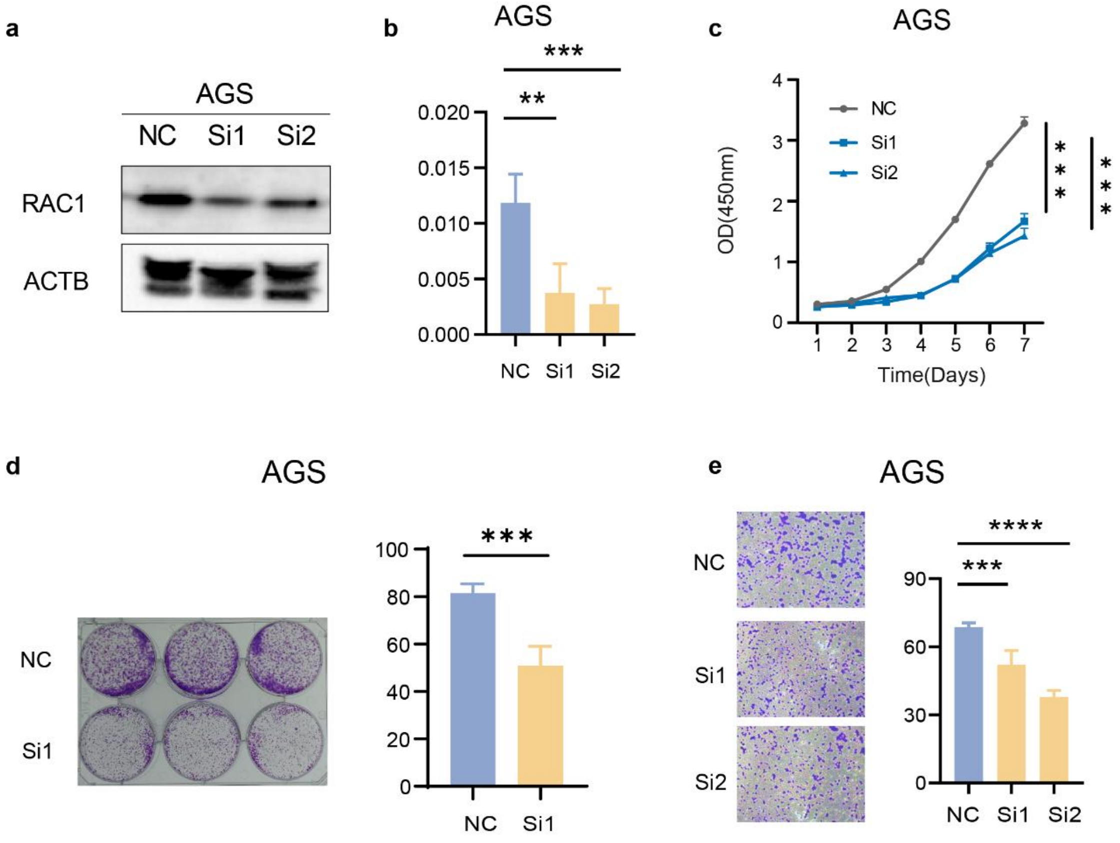

Figure S2 Knock down RAC1 in gastric cancer cells suppress cell proliferation and migration. (a-b) The knockdown efficiency of RAC1 in AGS cells at the protein level and RNA level. (c) The CCK-8 proliferation assay indicates that knockdown of RAC1 can significantly reduce the proliferation efficiency of AGS cell line. (d) The colony formation assay indicates that RAC1 knockdown significantly reduces colony formation. Representative images of colony formation assays (left) and quantification of the colony numbers (right) are shown. (e) The transwell assay indicates that RAC1 knockdown significantly reduces the migratory ability of AGS cell line. Representative images of transwell migration assays (left) and quantification of the migrated cells (right) are shown. (* $p < 0.05$, ** $p < 0.01$, *** $p < 0.001$, **** $p < 0.0001$).

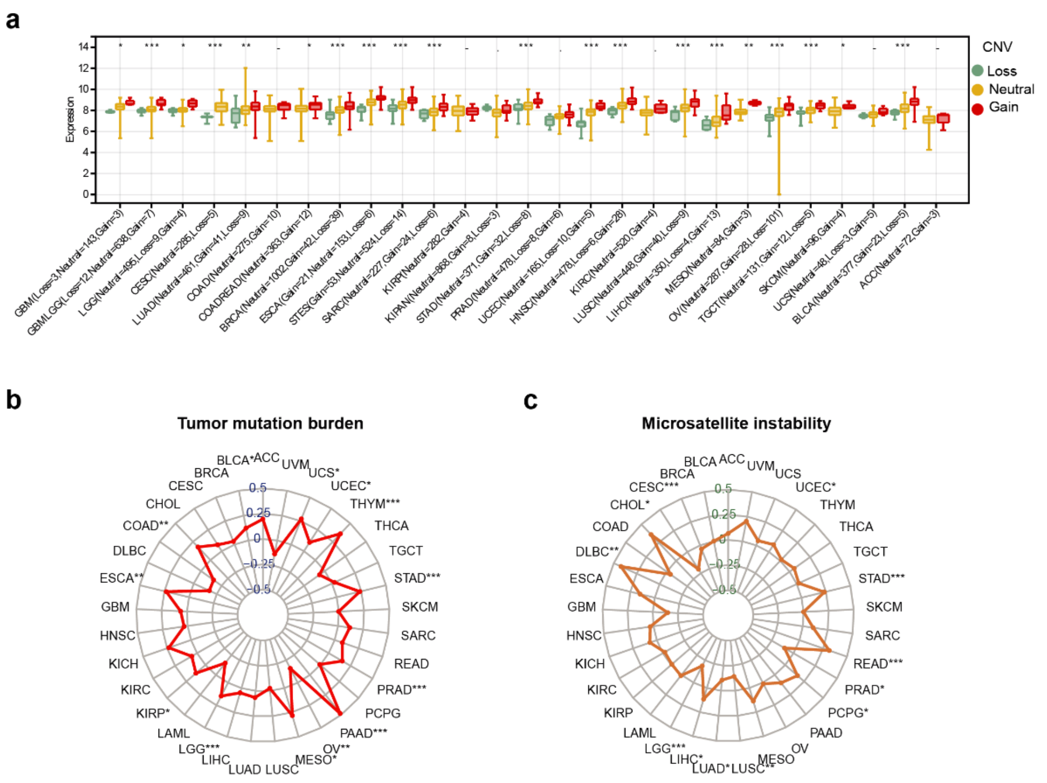

Figure S3 The correlation between RAC1 expression and CNV status, TMB and MSI at pan-cancer level. (a) The box clarifies that the expression level of RAC1 is related to the CNV status. (b-c) The radar chart illustrates the correlation between RAC1 expression and TMB and MSI at the pan-cancer level. (* $p < 0.05$, ** $p < 0.01$, *** $p < 0.001$, **** $p < 0.0001$).

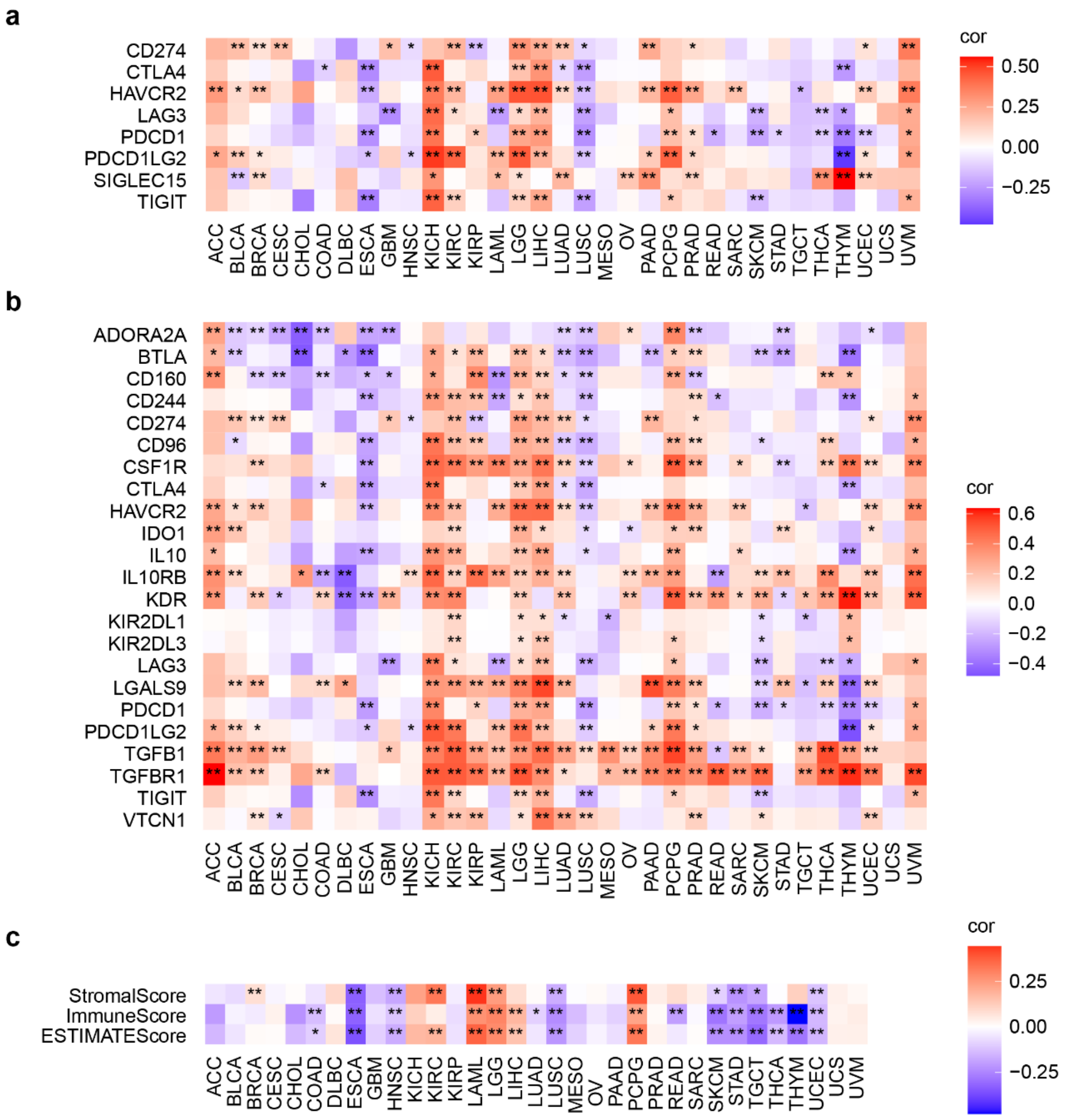

Figure S4 High expression of RAC1 is associated with immune checkpoint and inhibitory gene activity and a lower immune score. (a-b) The heatmap shows the association of RAC1 with immune checkpoint activity and immune inhibitory genes in various cancers. (c) The heatmap shows the results of ESTIMATE algorithm of RAC1. (* $p < 0.05$, ** $p < 0.01$, *** $p < 0.001$, **** $p < 0.0001$).

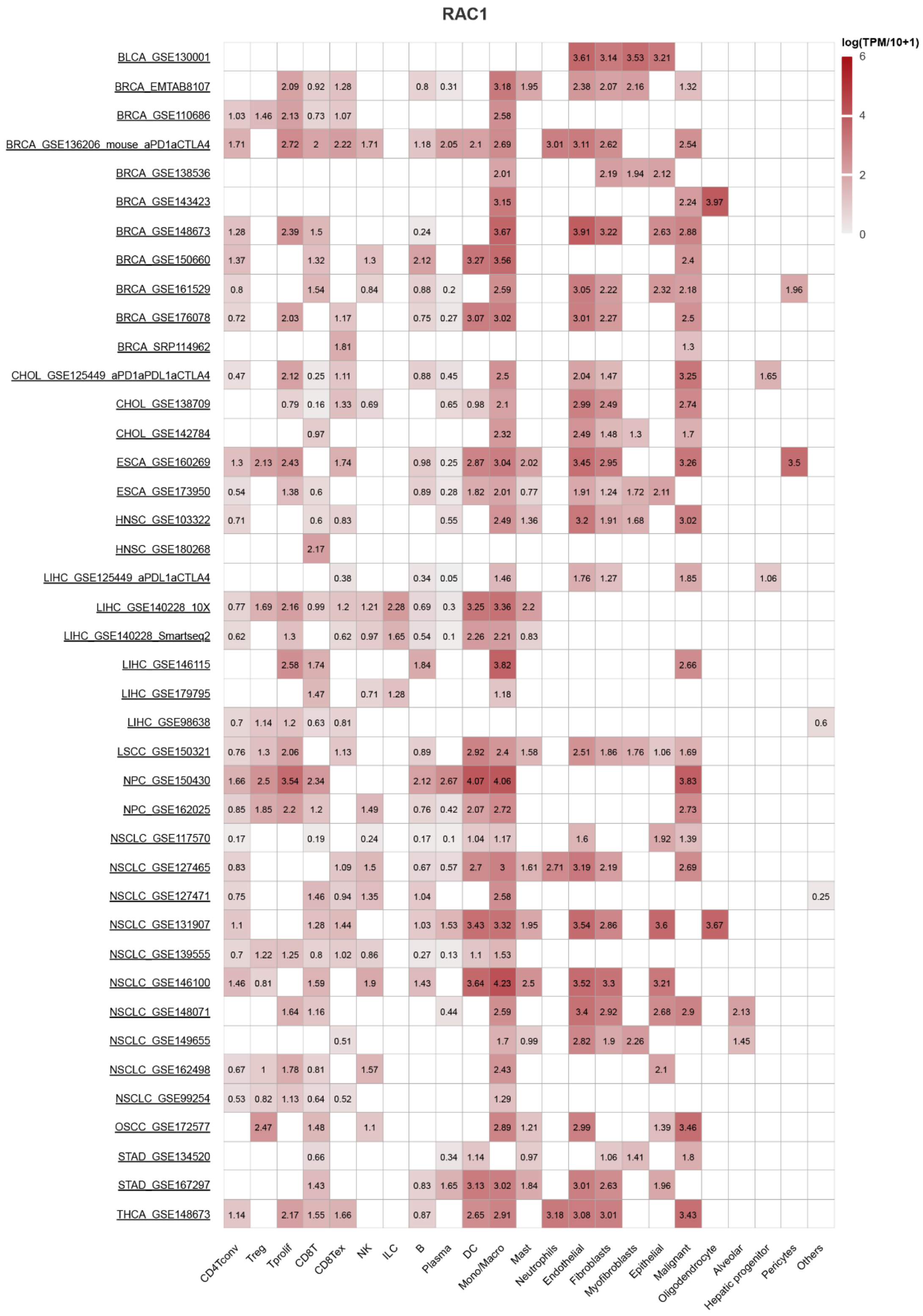

Figure S5 The single-cell analysis of RAC1 in pan-cancer from TISCH2 database.

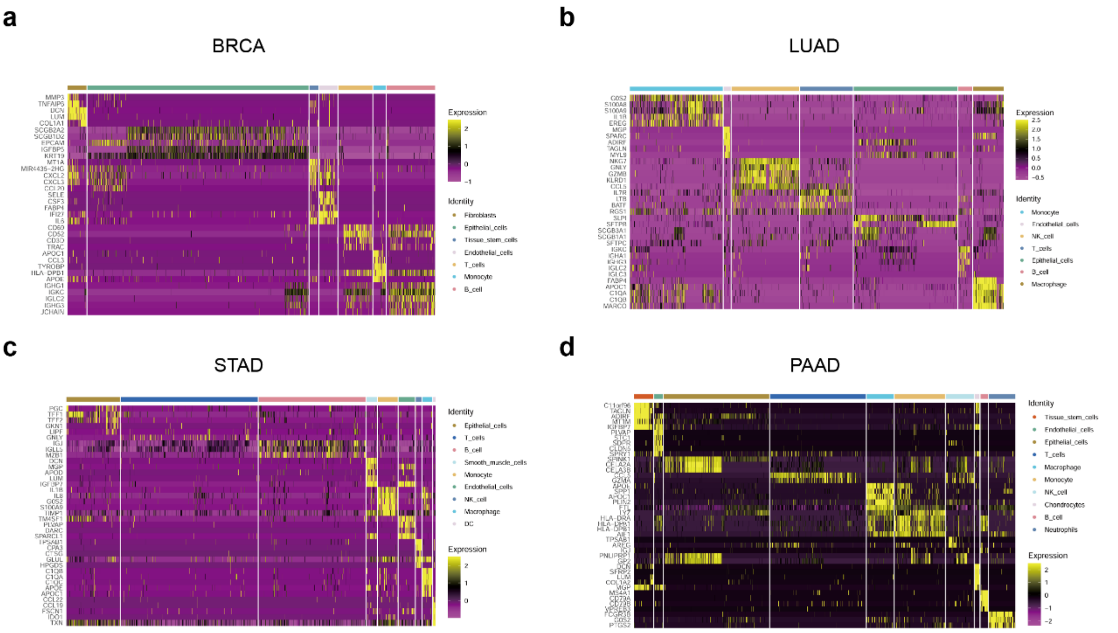

Figure S6 The heatmap displays the biological markers between single-cell subpopulations in (a)BRCA, (b)LUAD, (c)STAD and (s)PAAD.

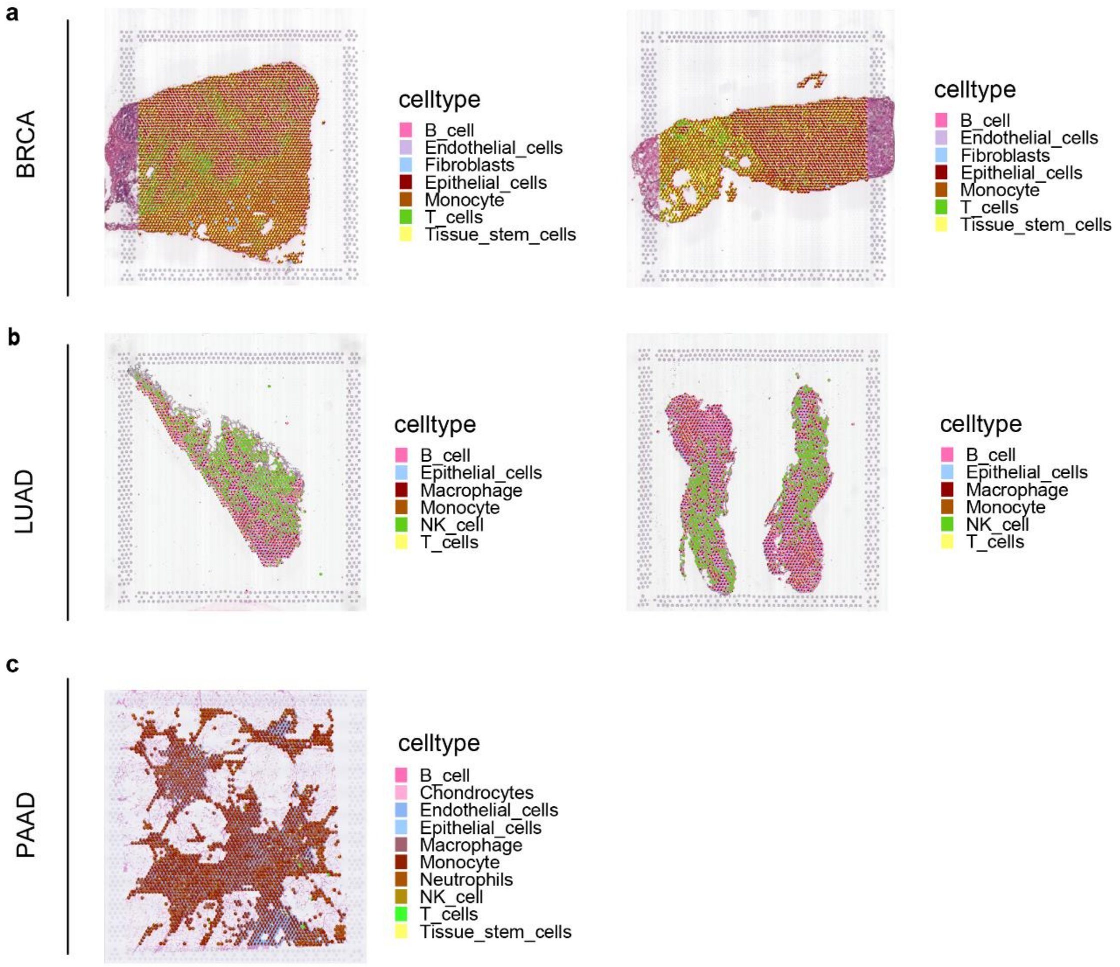

Figure S7 Deconvolution and annotation results of spatial transcriptomics data for (a) BRCA, (b) LUAD, and (c) PAAD using SPOTlight.
